# Assessment of community livelihood vulnerability to climate change in Vietnam: A case study of ethnic groups in Northern Upland Region

**DOI:** 10.1371/journal.pone.0330482

**Published:** 2025-09-08

**Authors:** Bui Thi Hoang Lan, Dinh Duc Truong, Tran Tho Dat, Bui Huy Quang, Nguyen Dieu Hang, Le Huy Huan

**Affiliations:** 1 Faculty of Environmental, Climate Change and Urban Studies, National Economics University (NEU), Hanoi, Vietnam; 2 School of Trade and International Economics, NEU, Hanoi, Vietnam; Yunnan University, CHINA

## Abstract

This study aims to assess the livelihood vulnerability to climate change of ethnic minority communities in Yen Bai province, a typical mountainous region in northern Vietnam. Utilizing the Livelihood Vulnerability Index (LVI) framework developed by Hahn et al. (2009), in combination with the IPCC vulnerability structure, the research analyzes eight components related to household characteristics, health, food, water, housing and productive land, social and financial networks, livelihood strategies, and exposure to climate shocks. Data were collected through a combination of desk study and survey with 480 households from two major ethnic groups: Tay and Thai.The results indicate that the Thai group has a higher overall LVI score (0.43) compared to the Tay group (0.37), reflecting greater livelihood vulnerability. The main factors contributing to this difference are limited livelihood diversification, lower educational attainment, weaker access to healthcare, and higher dependency on climate-sensitive resources. Although both groups are highly exposed to climate-related hazards such as flash floods, landslides, and droughts, the Thai group demonstrates greater sensitivity and lower adaptive capacity. This research contributes theoretically by adapting and refining the LVI framework to suit the context of upland ethnic communities, and practically by providing empirical evidence to inform climate adaptation policies. The study highlights the need for differentiated and context-specific strategies that prioritize ethnic minority communities with high vulnerability, focusing on improving education, livelihood diversification, healthcare access, and institutional support mechanisms.

## 1. Introduction

Climate change (CC) is one of the most serious challenges facing humanity in the 21^st^ century. CC causes long-term changes in global average temperatures, rainfall patterns, and extreme weather events such as droughts, floods, storms, and rising sea levels [1,2]. Although this is a global problem, developing countries are more severely affected due to weak adaptive capacity, limited infrastructure, and high dependence on agriculture and natural resources [3,4]. In these countries, CC threatens food security, household livelihoods, public health, and increases the risk of falling back into poverty [5,6]. Farmers in South Asia, sub-Saharan Africa, and Southeast Asia often lack access to climate-smart agricultural technologies, leaving agricultural production vulnerable to weather variability [7] In addition, cities in developing countries are at high risk of flooding due to poor infrastructure and rapid urbanization [8,9]. In addition, CC increases social inequality and places a disproportionate burden on vulnerable groups such as women, children, and ethnic minorities [10]. Strengthening adaptive capacity, promoting investment in green technology, and developing evidence-based adaptation policies are urgent measures that developing countries need to prioritize [8–11]. CC is not only an environmental issue but also a global development, security, and equity issue. Therefore, support from the international community, including climate finance and technology transfer, plays an important role in helping developing countries increase resilience and sustainable development in the context of rapidly changing climate [1,7,11].

Vietnam is one of the five countries most severely affected by CC, especially in vulnerable areas such as the northern mountainous region and the central coast [12,13]. According to the Ministry of Natural Resources and Environment (MONRE) (2020), the average annual temperature in Vietnam has increased by about 0.89°C during the period 1958–2018, with the frequency of extreme natural disasters increasing significantly [14]. Phenomena such as localized heavy rains, flash floods and landslides are occurring more frequently, causing serious damage. In 2020 alone, more than 100 landslides in the central region claimed the lives of more than 130 people and affected thousands of households [14]. Mountainous people – mainly ethnic minority households – are suffering heavy losses in agriculture: upland rice yields have fallen by 15–30% in the past 10 years due to erratic weather and prolonged drought [15,16]. In mountainous provinces such as Son La, Lai Chau and Ha Giang, more than 60% of households rely on traditional agriculture without active irrigation systems [17]. CC makes crop seasons unstable, spreads plant and animal diseases, reduces incomes and increases the risk of falling back into poverty. In addition, about 1.5 million mountain people are at risk of being affected by flash floods and landslides every year [14,18,19]. Despite many support programs, such as the Government’s Program 135 and internationally funded CC adaptation projects, people’s early warning capacity, disaster risk management, and access to information remain limited [20–22]. To enhance resilience, it is necessary to integrate indigenous knowledge with modern science, and invest in sustainable livelihood development, climate infrastructure, and social security systems in mountainous areas [23,24].

In recent decades, assessing livelihood vulnerability to CC has become a central topic in sustainable development studies, especially in developing countries in Asia – where people’s livelihoods are closely linked to agriculture, natural resources, and are greatly affected by climate [22,25–28]. One of the commonly used tools is the Livelihood Vulnerability Index (LVI) proposed by Hahn et al. (2009), which combines multiple indicators such as assets, income, health, access to information and adaptive capacity [29]. This method has been adapted and widely applied in many countries such as India, Bangladesh, Nepal, Thailand and Vietnam. For example, Pandey and Jha (2012) used LVI in India to assess farmers’ vulnerability to CC, showing that households with diversified income sources and better access to credit services have higher resilience. In Nepal, Sujakhu et al. (2019) combined LVI with GIS to identify highly vulnerable areas in coastal agriculture. Studies in Vietnam also show significant differences in vulnerability between population groups and ecological regions – with people in mountainous and coastal areas being more vulnerable due to their high dependence on natural resources and lack of access to climate information [30]. In addition to LVI, other methods are also applied such as SWOT analysis, PCA (Principal Component Analysis), AHP (Analytical Hierarchy Process) or DFID’s Livelihood Framework to measure impacts and propose appropriate intervention solutions [31–33]. The results of the studies all show that vulnerability is spatial, temporal and highly dependent on institutional capacity, community awareness and policy support. The use of composite indicators such as LVI is highly appreciated for its ability to quantitatively synthesize many aspects of livelihoods, but it also needs to be adjusted to the specific context of each country and region. In the context of complex CC developments, these studies are an important basis for planning effective, equitable and sustainable climate adaptation policies for vulnerable communities [34,30,32,35].

Globally, although studies on livelihood vulnerability to CC have increased, especially in developing countries in Asia, Africa and South America, there are still significant gaps in both theory and practice. In theory, most current studies are still based on the IPCC analytical framework (including three components: exposure, sensitivity and adaptive capacity) and composite indices such as LVI by Hahn et al. (2009) but there is a lack of integration between theories of livelihood resilience, institutional adaptive capacity and indigenous knowledge. Many studies continue to use old conceptual frameworks without expanding or updating them to reflect new forms of vulnerability in the context of complex, multidimensional, and regionally significant CC [25,27,34,33]. In practice, empirical studies still mainly focus on deltas, coastal areas, or areas with easy-to-collect data, while ethnic minority communities living in mountainous areas – which are highly exposed to climate risks – are rarely surveyed in depth [25,31]. Most international studies use quantitative assessment tools, but have not been closely combined with qualitative data, local knowledge, or socio-cultural indicators, especially in ethnically diverse areas. In addition, few studies have assessed the impact of institutional factors, such as supportive policies, the role of community organizations, or the quality of public services, in shaping adaptive capacity and reducing livelihood vulnerability. Another important gap is that most existing studies are static, whereas livelihood vulnerability is a continuous process that changes over time, depending on both the impacts of CC and the capacity of communities to respond and adapt [32,33]. Failure to incorporate time into assessments – for example, through seasonal cycles, ongoing CC, and changes in policy – results in assessments that are easily outdated or do not reflect reality. In Vietnam, although some recent studies have applied LVI in rural areas [15,17,30], most of them are still purely quantitative, lacking cultural and sociological depth, and have not explained the relationship between cultural diversity – livelihoods – climate vulnerability in ethnic minority communities. Therefore, this study aims to fill the gap by combining quantitative tools such as LVI with a participatory qualitative approach, and at the same time contribute to theory by proposing a conceptual model reflecting the characteristics of mountainous minority livelihoods in the context of CC.

This study aims to assess the livelihood vulnerability to CC of ethnic minority communities in Yen Bai, a typical mountainous province in northern Vietnam. The study uses the LVI developed by Hahn et al. (2009) and the IPCC as a framework to assess and analyze the vulnerability aspects of community livelihoods to CC. Theoretically, the study contributes to the extension and application of the LVI in the context of ethnic minority communities in mountainous areas of Vietnam – where there are distinct cultural, social and livelihood characteristics but is rarely surveyed in previous studies. The study also suggests the possibility of integrating local factors and indigenous knowledge into the theoretical framework for assessing vulnerability to CC. In practical terms, the study provides quantitative evidence and multidimensional analysis of livelihood vulnerability in Yen Bai province – a typical location in the Northern mountainous region. The research results can support policy makers and localities in designing appropriate, equitable and sustainable adaptation solutions for ethnic minority communities affected by CC.

The study is structured into 6 main parts: the introduction presents the context, gaps, objectives and significance of the study; part 2 presents the concepts and models for analyzing livelihood vulnerability due to CC; part 3 describes the research method, data collection and processing; part 4 presents the main research results; part 5 discusses the research results and compares them with previous studies, part 6 is the conclusions and implications drawn from the research results.

## 2. Conceptual framework and analytical model development

### 2.1. Conceptual framework

According to IPCC (2001), vulnerability to CC is the extent to which systems are susceptible to and unable to cope with the adverse impacts of CC, including climate variability and extremes. Accordingly, vulnerability is a function of exposure (E), sensitivity (S) and adaptive capacity (AC) [36].


V\ = \ f\ (E,\ S,\ AC)

**Fig 1:**
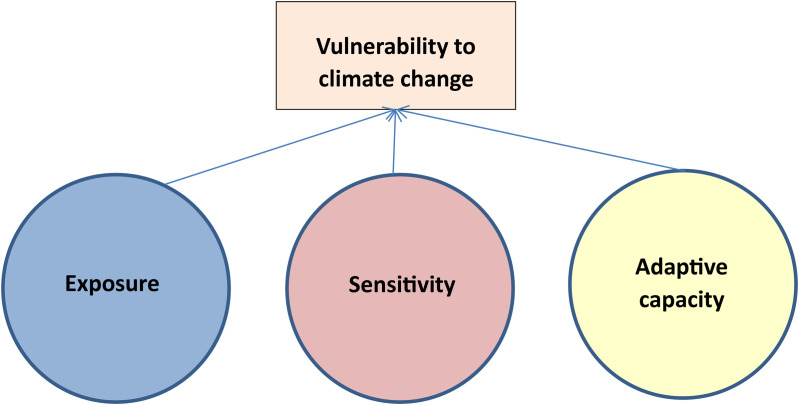
Livelihoods vulnerability to CC. Source: IPCC (2001).

In this function, exposure is the extent to which a livelihood system is exposed to the factors or agents of CC, such as increased temperature, altered rainfall patterns, droughts, floods, landslides or extreme weather events. Exposure reflects external risks that the community cannot control, but are a prerequisite for subsequent impacts. For example, a community living near a river or on a mountain slope will have a higher level of exposure to flash floods or landslides. Sensitivity reflects the extent to which a community’s livelihood is affected when exposed to climate factors, depending on internal characteristics such as: production structure, main source of livelihood, health status, dependence on natural resources, etc. Sensitivity shows the vulnerability from within the community. For example, a community that depends mainly on natural rain-fed agriculture will be more sensitive to drought than a community with a good irrigation system. Adaptive capacity is the ability of a community to adjust, cope, and recover from the impacts of CC, through factors such as: level of access to information, education level, financial resources, infrastructure, and support from policies and social networks; tte higher the adaptive capacity, the lower the vulnerability. For example, communities with skills to diversify their livelihoods, access credit, or participate in government support programs are often more resilient (Fig 1).

In recent years, methods for assessing livelihood vulnerability to CC using comprehensive indices have been widely applied in many developing countries [37,38]. This approach helps to synthesize a variety of qualitative and quantitative indicators into a single index or a group of indicators, thereby comprehensively reflecting the vulnerability level of a community or region [22,31,38]. Common methods include comprehensive LVI, LVI-IPCC index (integrating the theoretical framework of IPCC), Social Vulnerability Index (SoVI), or GIS-based vulnerability index [38,39]. Among them, LVI developed by Hahn et al. (2009) is one of the most widely used tools due to its flexibility, adaptability to local conditions and ability to quantify qualitative aspects of livelihoods. This LVI integrates three main components: exposure, sensitivity and adaptive capacity – in line with the IPCC assessment framework. This method also allows for data standardization, division into thematic indicator groups (such as health, education, resources, climate, etc.) and calculation of a composite index. In addition, LVI can also be extended to the LVI-IPCC version to assess according to the components of climate vulnerability [40]. This study chose LVI as the assessment method because it is suitable for the characteristics of ethnic minority areas in the mountainous areas of Northern Vietnam, where data is limited and requires a method that can both quantify and reflect social and cultural characteristics. The application of LVI helps to specifically reflect the vulnerability aspects of community livelihoods in a systematic way, while facilitating comparisons between ethnic groups, geographical regions or over time. Furthermore, this method has been verified in many studies in developing nations such as in India [25], Nepal [26],Ghana [34], and Trinidad and Tobago [24], showing high applicability in the context of developing regions with similar terrain and population conditions to Vietnam.

### 2.2. Analytical model development

This paper applied the LVI model by Hahn et al. (2009) and IPCC for assessing livelihood vulnerability to CC in Yen Bai. The LVI proposed by Hahn et al. (2009) is structured according to a composite index model, in which specific indicators are grouped into 7 major components, each group reflecting an aspect of livelihood vulnerability. These groups include: socio-demographic profile, livelihood strategies, health, food, water access, social networks, exposure to natural disasters and climate variability. These groups of indicators are then standardized to the same scale (0–1), averaged by group, and then aggregated into a general LVI index for each household or community. In the LVI-IPCC version, these index groups are also divided into three main components according to the IPCC: exposure, sensitivity and adaptive capacity [29].

In the context of the study in the mountainous ethnic minority area of Yen Bai province, the demographic characteristics of households play a very important role but can affect vulnerability from two different directions, so in this study, we separate the “demographic profile” index group according to the original structure of Hahn et al. (2009) into two separate component groups: household characteristics and housing and productive land. First, the group of household characteristics (including the number of dependents, education level of the household head, production experience, gender of the household head, etc.) has a clear influence on the adaptive capacity of the household. Households with low dependency ratio, household heads with high education and production experience are often able to make more effective decisions, access information and adapt to CC better [25–27,29,31,32]. Therefore, this group of indicators is included in the adaptive capacity component in the study. Second, the group of indicators on housing and productive land such as: housing quality, agricultural land area, land ownership status, etc. clearly reflects the level of physical vulnerability of households when extreme climate events or natural disasters occur. Households living in temporary houses, or having limited land area for cultivation are often more vulnerable to climate risks, so these are suitable factors to be included in the sensitivity group in the LVI-IPCC assessment framework [25,38,39].

Separating these two groups of indicators not only helps to increase the accuracy in reflecting each component of vulnerability, but also suits the terrain and living conditions of the mountainous people, where housing conditions and access to productive land differ significantly among ethnic groups and directly affect their livelihoods. At the same time, this adjustment also demonstrates the flexibility and local adaptability of the LVI model, in line with recommendations in recent studies that the LVI index should be adjusted to reflect the characteristics of each region or specific population group. Table 1 shows the LVI component indices in this study.

**Table 1:** LVI components in the study.

	Main components
Adaptive capacity	Household characteristics
	Livelihood strategies
	Social and finacial network
Sensitivity	Health
	Food
	Water access
	Housing and productive land
Exposure	Disaster and CC

Source: Adapted from literature (2024).

Below is the LVI calculation formula developed according to the approach of Hahn et al. (2009), adjusted to suit the study of ethnic minorities in the mountainous areas of Yen Bai province:

*Sub-index normalization*

Each sub-index is normalized to the same scale from 0 to 1 according to the formula:


$$S_{i}=\frac{X_{i}-X_{min}}{X_{max}-X_{min}}$$

- S_i_: standardized value of sub-index i
- X_i_: actual value of sub-index i
- Xmin, Xmax: minimum and maximum values of sub-index in the entire survey sample

*Main component indices calculation*

Each main component index group (health, food, housing...) is averaged from the standardized sub-indices:


$$M_{j}=\frac{\sum_{i=1}^{n}S_{i}}{n}$$

- M_j_: mean value of the j^th^ main component index group
- n: number of sub-indices in that group
- S_i_: standardized sub-indices

***Comprehensive LVI calculation***


$$LVI=\sum_{j=1}^{m}W_{j}M_{j}$$

- LVI: comprehensive livelihood vulnerability index
- M_j_: mean value of the j^th^ main component index group
- W_j_: weight of the jth main component index group (in this study the weights for the groups were assumed to be equal.)
- m: total number of main component index groups

According to the above calculation, the value of LVI ranges from 0 to 1, the closer to 1, the higher the level of vulnerability to climate change.

Another estimated version of LVI in this study is the LVI–IPCC, in which instead of aggregating the main factors into the LVI, they are divided into three groups of vulnerability factors based on exposure, sensitivity, and adaptive capacity to CC.


LVI−IPCC=(E−AC)×S

- E: exposure to climate risks
- AC: adaptive capacity
- S: sensitivity to climate risks

$$E=\frac{\sum_{j=1}^{k}M_{j}}{k}S=\frac{\sum_{j=1}^{l}M_{j}}{l}AC=\frac{\sum_{j=1}^{m}M_{j}}{m}$$

- k: number of index groups in exposure
- l: number of index groups in sensitivity
- m: number of index groups in adaptive capacity

Accordingly, LVI-IPCC > 0 means that the livelihood is vulnerable to climate change, the closer to 1, the higher the vulnerability, LVI-IPCC < 0 means that the livelihood is less vulnerable.

## 3. Methodology

### 3.2. Desk study

In the first phase, the research team conducted a desk study to develop a theoretical basis and analytical framework for the study. The reviewed documents included domestic and foreign academic works on livelihood vulnerability, LVI and LVI-IPCC indexes, previous studies on livelihoods and climate change adaptation in ethnic minority areas, as well as policy reports, meteorological and hydrological data and relevant local information. This phase helped the research team identify the main components of the LVI index (such as: household characteristics, health, livelihoods, food, water, land, social networks and natural disasters), and referenced the index structure according to Hahn et al. (2009) as a basis for the research design.

### 3.3. Expert and community consultation for survey design

As part of the expert and community consultation process to ensure contextual relevance and cultural appropriateness, a Focus Group Discussion (FGD) was conducted to develop and finalize the component indicators in the LVI framework, and at the same time helping to develop a survey toolkit suitable for the specific context of ethnic minority communities in mountainous areas in Yen Bai province. The FGD was conducted after the literature review phase, to ensure that the proposed indicators are not only theoretically based but also closely reflect the realities of local livelihoods and risks. Specifically, one FGD was conducted with the main subjects being village heads, local authorities and people. We selected local people in Muong Lai commune to participate in the FGD. Of which, 8 household heads were randomly selected from all 8 villages in the commune, including 4 Tay households and 4 Thai households. In addition, the vice chairman of the commune and the head of a village were also invited to participate in the FGD. The main contents of the FGD include: (i) Discussing the major components of the LVI (e.g., health, food, water, land, livelihoods, social networks, natural disasters, etc.) and assessing their suitability to local conditions; (ii) Developing and adjusting sub-indicators through open-ended questions and answers, sharing experiences, and assessing community risks and resources. (iii) Assessing people’s language and understanding of the concepts in the survey (e.g., “water shortage”, “food insecurity”, “livelihood diversity”, “social support”), thereby editing the wording to make it easier to understand and suitable for local culture; (iv) Identifying some quantitative thresholds and classification criteria, for example: how many months of food shortage is considered food insecurity, or how far is considered “difficult to access water” in the mountainous context. Through the FGD, the research team collected important practical information, helping to adjust the LVI index and structure to better suit the local community. Some notable adjustments include: separating the “Housing and productive land” index group from “Household characteristics” to more clearly reflect the role of physical conditions on sensitivity; adding indicators on community support and the role of indigenous knowledge in adaptation; and more clearly defining the impact of common local natural disasters (hail, flash floods, mid-season drought). The results from the FGD were then used to design a questionnaire for the household survey.

### 3.4. Pilot survey

After finalizing the questionnaire based on the literature review and FDG results, the research team conducted a pilot survey to test the clarity, appropriateness, and feasibility of the survey tool in practice. This was an important step to ensure that the questionnaire was not only valuable in content but also easy to understand, easy to use, and appropriate to the cognitive level and cultural context of the local ethnic minority community. The pilot survey was conducted in a village with a total of 20 households from the Tay and Thai ethnic groups randomly selected. The survey forms were administered directly by the surveyors under the supervision of the research team, with an average duration of 35–40 minutes per household. The pilot survey helped identify some problems in the questionnaire design, including: some technical terms such as “livelihood diversity”, “sensitivity level”, “informal support” are difficult for people to understand → need to change the wording to simpler language or explain with specific examples. In addition, some quantitative indicators (such as distance to water sources, number of natural disasters) need to be redefined in specific units of measurement to make it easier for people to answer and ensure consistency when synthesizing data. After the pilot survey, the research team adjusted the content and structure of the questionnaire, including rearranging the order of the sections according to the logic of the actual experiences of the households, and completing the instructions for the interviewers on how to record, handle situations and make special notes. The results from the pilot survey not only helped improve the quality of the survey instrument, but also helped the research team estimate interview time, test interviewer skills, and detect potential risks during the formal data collection process.

### 3.5. LVI component indices

From the results of FGD, pilot survey combined with LVI models based on literature [22,25,26,31,32,34,35], the study built component and sub-component indices to calculate LVI for study site inYen Bai (Table 2).

**Table 2:** LVI component and sub- components.

Main and sub – components		Descriptions	Unit
**Household characteristics**	1.1.Dependency Ratio	Population <15 years old and >60 years old/ Total household population	%
	1.2.Household head education	Percenntage of household heads completed at least primary school	%
	1.3.% of female household heads	Percentage of female household heads	%
	1.4.% of household heads with incomplete primary education	Percentage of households with a household head with incomplete primary education.	%
**Livelihood strategies**	2.1. Percentage of households primarily dependent on agriculture	Percentage of households considering agriculture as the primary source of livelihood	%
	2.2. Percentage of households with no members working outside the village	Percentage of households with at least one family member working in another commune/town/city/province	%
	2.3. Percentage of households with no sources of income other than agriculture	Percentage of households with family members engaged in occupations other than farming, animal husbandry, or forest and stream product harvesting as a primary source of income	%
	2.4. Average agricultural livelihood diversity index	Inverse proportion of the number of types of agricultural/forestry livelihoods in the family (only households primarily reliant on agriculture/forestry)	0–1
**Social and financial network**	3.1. Percentage of households with no access to information sources	Percentage of households without at least one form of information communication technology, such as TV, radio, telephone, or internet	%
	3.2. Diversity index of information sources	Inverse ratio of the sum of information communication technologies (number of means+ 1).	0–1
	3.3. Percentage of households receiving assistance from government agencies in the past 12 months	Percentage of households that received assistance from government authorities in the past 12 months	%
	3.4. Average proportion of receiving: giving (aid)	Ratio (sum of types of assistance received in the past month + 1 to the sum of types of assistance provided to other households in the past month + 1)	
	3.5. Average proportion of borrowing: lending money within the community	Ratio (sum of money borrowed + 1 to the sum of money lent +1) within the community (relatives, friends, neighbors) in the past month (if households borrowed but did not lend money, the ratio is calculated as 2/1 = 2; if they did not borrow but lent money, the ratio is calculated as 1/2 = 0.5).	
	3.6. Average proportion of borrowing: lending money from/to banks at present	Ratio of the sum of money borrowed from banks + 1 to the sum of money deposited in banks + 1 at the current time.	
	3.7. Average distance to the town center	Average distance from households to the town center (km) – obtained from village leaders	km
**Health**	4.1. Percentage of households with family members suffering from chronic illnesses	Percentage of households with at least one family member suffering from chronic diseases such as diabetes, heart disease, asthma, and others	%
	4.2. Percentage of households with members in need of care	Percentage of households with family members who require daily care (elderly, children, disabled individuals)	%
	4.3. Percentage of households with family members taking sick leave from work/school in the past two weeks due to illness	Percentage of households reporting that at least one family member had to take sick leave or miss school in the past two weeks due to illness	%
	4.4. Average distance to the nearest healthcare facility	Average distance to the local community health center or clinic (km)	km
**Food**	5.1. Percentage of households primarily relying on self-produced food and agricultural products as their main source	Percentage of households primarily relying on family crops and livestock for their food	%
	5.2. Average number of months experiencing food shortage.	Number of months the family experienced difficulty in earning enough food	month
	5.3. Average diversity index of crops	Inverse ratio of the number of crops grown (only for households engaged in agriculture)	0–1
	5.4. Percentage of households mainly dependent on foraging in the forest for their food.	Percentage of households reporting that the family’s main food source is from foraging in the forest	%
	5.5. Percentage of households without food reserves for the next planting season	Percentage of households without food reserves for the next planting season (excluding sales)	%
**Water access**	6.1. Percentage of households primarily using natural sources for domestic water supply	The proportion of households using natural sources such as ponds, lakes, rivers, or streams as their main source of domestic water supply	%
	6.2. Average travel time to the main water source for household use	The average time it takes to travel from home to the primary water source used for household	minutes
	6.3. Percentage of households with insufficient water supply for year-round use	The percentage of households reporting that they do not have enough water for year-round domestic use	%
**Housing and productive land**	7.1. Percentage of households with non-durable housing susceptible to windstorms and hail	The percentage of households with non-durable housing (vulnerable to roof damage and collapse due to strong winds, hail, and heavy rain)	%
	7.2. Percentage of households with low-lying land susceptible to flooding	The percentage of households located in low-lying areas, depressions, or near rivers and streams that are susceptible to flooding during the rainy season	%
	7.3. Percentage of households with homes located in areas prone to landslide	The percentage of households situated near hillsides or sloping terrain that are prone to landslides during the rainy season	%
	7.4. Average distance to the main agricultural production land	The average distance from homes to the main agricultural production land of the household	km
	7.5. Average area of agricultural land susceptible to drought	The average extent of agricultural land regularly affected by drought	ha/household
	7.6. Average area of agricultural land susceptible to flooding	The average extent of agricultural land frequently inundated by flooding	ha/household
**Disaster and CC**	8.1. Percentage of households experiencing property damage due to natural disasters in the past 7 years	Percentage of households experiencing property damage due to natural disasters in the past 7 years	%
	8.2. Percentage of households with individuals injured or killed due to natural disasters in the past 7 years	Percentage of households with individuals injured or killed due to natural disasters in the past 7 years	%

Source: Proposed in study (2023).

### 3.6. Sampling and data collection

In this phase, the survey sample was calculated and the households participating in the interviews were identified. To calculate the realiable sample size, following formula was used [40]:


$$n=\frac{N}{1+N\times \varepsilon^{2}}$$

n is sample size, N is total households, ε is error. As the total household in Muong Lai commune is 1,875 and with ε = 0.05, the reliable sample size was 398. In 2021, Muong Lai commune restructured its administrative units, dividing it into 8 villages. For this study, the authors selected all 8 villages for collecting information. The survey team prepared 480 questionnaires (60 per village), and households were randomly selected for the survey. In each village, the research team collected a list of households from the commune People’s Committee (local government), then randomly drew households from the list for the survey. Households were approached in the late afternoon when the head of the household is usually present after work. If a household was absent, the next household on the list was interviewed. An interview usually lasts 30–40 minutes, with questions focusing on five main areas: (i) general information about the household, (ii) household awareness of natural disasters and their impacts on livelihoods, (iii) household livelihood activities, (iv) health status, food and water sources, and (v) participation in government support programs, participation in local associations, assets and production equipment. All participants must give written informed consent to participate in the study. Before answering the questions, the interviewee was specifically introduced to the objectives of the interview and the purpose of the research. At the same time, they were asked if they agreed and were willing to participate in the interview. All respondents agreed and were willing to participate and ticked into the options of willing to participate in the questionnaires. After conducting the survey, 456 valid results were collected, summarized, and used for statistics in the study (Table 3).

**Table 3:** Villages and number of valid questionnaires.

No.	Village	Ethnic group	Valid questionnaires
1	Na Chao	Tay	57
2	Na Chen	Tay	57
3	Na Va	Tay	56
4	Na Chua	Tay	58
5	Na Cay	Thai	59
6	Na Bo	Thai	60
7	Na Khoang	Thai	54
8	Na Ngam	Thai	55
**Total**			**456**

Source: Study survey (2023).

The study also collected secondary data on natural disasters and CC in Yen Bai and other socio-economic information at Vietnam General Department of Meteorology, Hydrology and Climate Change, Yen Bai Provincial Statistics Department and the Vietnam General Statistics Office (GSO).

### 3.7. Data analysis

All collected data were processed and analyzed using SPSS 26.0. Descriptive statistical methods were first employed to summarize the demographic, social, and livelihood characteristics of the surveyed ethnic minority households. These included the calculation of frequencies, percentages, means, standard deviations, and ranges for each sub-component of LVI. The purpose of this initial step was to provide a general overview of the socio-economic profiles and vulnerability dimensions across the two ethnic groups (Tay and Thai), allowing for a clearer understanding of patterns in exposure, sensitivity, and adaptive capacity. Visualizations, including bar charts and summary tables, were used to present these findings.

In order to deepen the analytical rigor of the study and support the claim of a quantitative approach, we also applied inferential statistical tests to assess the significance of observed differences between the two ethnic groups. Specifically, Student’s t-tests were used for continuous variables (e.g., average age, dependency ratio, and distance to services), testing whether the mean values between Tay and Thai groups were significantly different. Meanwhile, for categorical or proportion-based variables (e.g., percentage of households dependent on agriculture, use of natural water sources, receipt of government support), Chi-square (χ²) tests were employed to determine whether the distribution of responses differed significantly between the groups. All tests were conducted using a 95% confidence level (*α* = 0.05), and a p-value of less than 0.05 was considered statistically significant. These inferential analyses help validate whether the observed disparities in vulnerability indicators are likely to reflect broader group-level patterns rather than being due to chance.

## 4. Results

### 4.1. Study area

**Fig 2:**
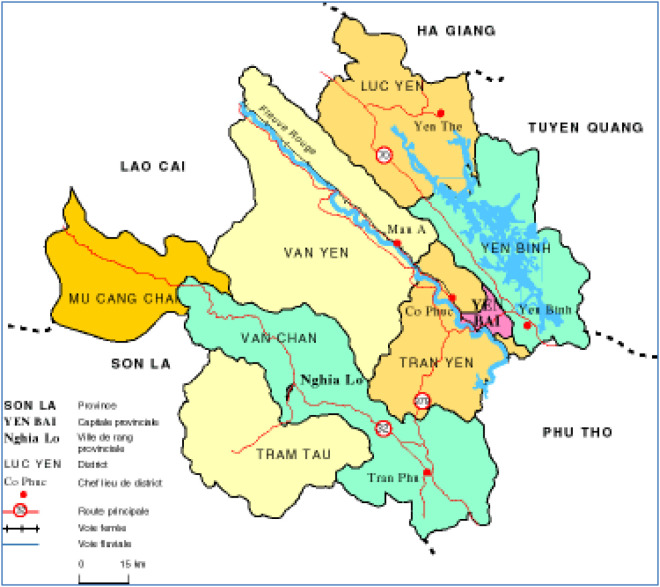
Yen Bai province. Source: Adapted from https://www.angelfire.com/co/hongnam/vnmap/yenbai.html (2025).

Yen Bai is a mountainous province located deep in the interior of Northern Vietnam, between the Northeast and Northwest regions. The North borders Lao Cai province, the South borders Phu Tho province, the East borders Ha Giang and Tuyen Quang provinces and the West borders Son La province. Yen Bai has 9 administrative units (1 city, 1 town and 7 districts) with a total of 180 communes, wards and towns, including 70 highland communes [41,42] (Fig 2).

In this study, Luc Yen district was selected because it is a typical district in terms of natural and socio-economic conditions of Yen Bai. Naturally, it is a land divided by main mountain ranges, creating a diverse terrain including mountain ranges, valleys and small streams, cool climate. Socially, this is home to ethnic minority groups in the northern upland of Vietnam. Specifically, thís is a mountainous district located in the northeastern part of the province, bordering the provinces of Lao Cai, Ha Giang, and Tuyen Quang. It has a population of over 100,000 people, consisting of ethnic groups living in 24 communes and townships. This is one of the major rice-producing regions in Yen Bai province [41]. The district is home to 2 main ethnic groups: Tay and Thai, with the Tay community constituting the majority of the population. The Thai people typically reside in highland areas, far from the district center and communes, while the other ethnic groups predominantly inhabit lowland areas (valleys) [43,44]. Faced with population pressure, increasing demands for a better quality of life, and the depletion of resources coupled with limited access, the majority of the highland residents are increasingly grappling with livelihood challenges, including those posed by CC [41]. In recent years, Luc Yen has been notably affected by natural disasters, experiencing prolonged droughts and abnormally heavy rainfall with thunderstorms [42,43]. These climate-related events have not only impacted agricultural production but have also had significant consequences on the daily lives and activities of the local population. Due to its geographical location within a highland region and a significant average elevation of approximately 600 meters, the communes harbor numerous CC vulnerabilities. Concerning livelihood sources, the local residents predominantly depend on crop cultivation, animal husbandry, and forestry. Key annual crops include rice and maize, with maize serving as the primary cash crop [43]. Currently, many areas that were previously dedicated to maize cultivation are gradually shifting towards sugarcane and fruit tree cultivation, driven by market dynamics and land-related considerations. Alterations in agricultural seasons and crop yields have led to food supply instability and reduced income for the local population. Also, the depletion of water resources and natural assets, including forests, streams, and vegetative cover, has affected traditional livelihood sources such as animal husbandry and forestry [43,44].

Within the district, Muong Lai commune was selected to collect information. The commune is located approximately 13 kilometers north of Yen The town, the central hub of Luc Yen district, with an average elevation ranging from 400 meters to 1,500 meters above sea level. Over 80% of the commune’s total land area consists of hilly terrain, encompassing rolling hills, sloping hills, and high mountain ranges. The natural land area of the entire commune covers 41.27 square kilometers, primarily characterized as agricultural and forestry land (constituting 86%). By 2023, the commune was home to 1,875 households, encompassing 8,270 inhabitants from 11 ethnic groups. Among these, the Tay People make up more than 60%, followed by the Thai People (38%). The Kinh ethnic group represents less than 1% of the commune’s total population [41,42].

Regarding CC, over the past 3 decades (1990–2020), the average annual temperature of Yen Bai province has increased about 0.4^0^C to 0.6^0^C; the average annual rainfall has gradually decreased, fluctuating between 1,750 mm and 1,850 mm. Extreme climate phenomena related to temperature and rainfall have appeared in the province such as increased cold air, hot weather, snow, ice, hail, and unseasonal heavy rain. The number of cold spells has decreased significantly (from an average of 29 cold spells per year in the decades from 1981 to 2010 to only 15–16 cold spells per year in the decade from 2011 to 2020 [42]. However, the number of cold spells has fluctuated strongly from year to year, with record-breaking cold spells and cold spells with quite low temperatures. During the rainy season, prolonged and heavy rains accompanied by whirlwinds and sometimes hailstorms cause floods, inundation, flash floods, and landslides that damage crops, lives, and property of people in riverside and stream areas. During the 2011–2020 periods, weather conditions were unusual; extreme temperature-related phenomena such as cold air, severe cold, and frost occurred earlier than the average of many years; in the winter of 2021–2022, severe cold and widespread cold did not last long, but temperatures reached the lowest values in the past 40 years. In addition, there were quite strong cold spells, causing moderate and heavy rains rarely seen in the observation data series in the middle of the dry season [42,43].

### 4.2. LVI results

#### 4.2.1. *Socio-demographic profile.*

Table 4 presents the basic demographic characteristics of households belonging to the two ethnic groups Tay and Thai surveyed. This information is important in analyzing adaptive capacity and livelihood vulnerability to climate change because it reflects the potential of labor resources, dependency level, educational level and social conditions of the household.

**Table 4:** Demographic profile of Tay and Thai groups.

Socio-demographic profile		Unit	Tay group	Thai group
**Gender**		%		
Male			74	73
Female			26	27
**Household head’s age**				
Male	<25	%	6	9
	25–40		45	53
	41–55		18	20
	>55		31	18
Female	<25		4	20
	25–40		47	53
	41–55		29	27
	>55		20	0
**Number of members**		people/household	4.8 ± 1.6	5.2 ± 1.6
**Number of members < 15 years old**			1.5 ± 0.7	2.2 ± 0.9
**Number of members > 60 years old**			0.5 ± 0.8	0.4 ± 0.7
**Household head’s education level**				
**Male household head**				
Not going to school, unable to read and write		%	10	30
Not going to school, able to read and write			5	6
Incompleted grades 5			33	32
Completed grades 5–9			47	22
Completed grades 9–12			5	10
**Female household head**				
Not going to school, unable to read and write		%	35	91
Not going to school, able to read and write			9	2
Incompleted grades 5			56	7
Completed grades 5–9			0	0
Completed grades 9–12			0	0

Source: Fieldwork (2023).

In terms of household size, Thai households have an average of 5.2 people/household, higher than the Tay group with 4.8 people/household. Although the difference is not large, this partly reflects the young population structure and multi-generational households common in the Thai group, which may increase the burden of dependency in the household. The proportion of children under 15 years old in the Thai group is 2.2 people/household, compared to 1.5 people/household in the Tay group, indicating that the Thai group has a younger population but also has a higher risk of having to pay for care and education – a factor that can reduce the ability to accumulate and invest in adaptation. In terms of household head gender, the proportion of households headed by men is dominant in both groups, with 86.3% in the Tay group and 91.7% in the Thai group. The proportion of female-headed households in the Tay group is 13.7%, higher than the Thai group (8.3%), partly reflecting the higher social participation and decision-making capacity of women in the Tay group. The educational level of the household head is an important indicator of the capacity to access information and adapt. The results show that the Tay group has a significantly higher literacy rate. Specifically, 64.7% of Tay household heads had completed primary school, 19.6% had completed secondary school, and only 4.9% were illiterate. Meanwhile, in the Thai group, the rate of illiterate people was up to 33.3%, only 47.2% had completed primary school, and only 5.6% had completed secondary school or higher. In terms of production experience, the Tay group had an average agricultural experience of 23 years, 19 years higher than the Thai group. This shows that the Tay group has a better experience base in traditional agricultural production – a factor that can contribute to increased adaptive capacity, especially in unstable production conditions due to climate change. Finally, the rate of households with disabled or elderly people living together is a factor that increases vulnerability. The Thai group has a higher proportion of households with elderly people over 65 years old (21.5%) than the Tay group (17.8%), while the proportion of households with disabled people is almost the same (about 6–7%).

The above characteristics show that the Thai ethnic group has more disadvantageous demographic conditions than the Tay group, especially in terms of education level, dependency ratio and access to basic services. These factors play an important role in forming the adaptive capacity of households – a key component in the LVI structure.

Table 5 provides details of the component indicators that make up the “household characteristics” index group in the LVI, reflecting the level of vulnerability related to demographic factors and basic capacity of Tay and Thai households. This component index in the Thai group is 0.45, higher than that of the Tay group at 0.41, indicating that the Thai group has a higher level of vulnerability in the demographic aspect. Specifically, the first indicator – the dependency ratio (number of people under 15 and over 60 years old in the total population) – in the Thai group is 46.7%, higher than that of the Tay group at 38.2%. This reflects higher economic and social pressure on the Thai group, as the number of people not directly involved in production is larger, reducing the proportion of active labor in the household. In addition, this group is also highly vulnerable in the context of natural disasters or epidemics. The indicator of household head education shows a clear differentiation: while 64.7% of Tay household heads completed at least primary school, only 47.2% of Thai household heads achieved this level. Notably, the rate of illiterate household heads in the Thai group is 33.3%, nearly 3 times higher than that of the Tay group (11.7%). Low education level not only affects production management capacity and access to agricultural techniques, but also reduces the ability to receive climate warning information and support policies from the State. The proportion of female-headed households is a factor reflecting the level of women’s participation in household management and livelihood decisions. The Tay group has 13.7% of female-headed households, higher than the Thai group at 8.3%. However, among female-headed households, the literacy rate in the Tay group is 61.2%, while in the Thai group it is 71.0%. This reflects that Thai women are not only disadvantaged in terms of decision-making power but also seriously disadvantaged in terms of learning ability and access to resources. Length of residence in the locality and production experience are also indirect indicators of understanding of natural conditions and local social networks. The Tay group has an average length of residence in the locality of 26.5 years and an average agricultural production experience of 23 years, while the Thai group has 21.7 years and 19 years, respectively. This difference suggests that the Tay group has a better foundation of local experience and knowledge, which can help them adapt more quickly to environmental fluctuations or changes in farming techniques.

**Table 5:** LVI score for household characteristics.

Sub-components	Unit	Tay group		Thai group		Min	Max
		Real Value	Standardized Value	Real Value	Standardized Value		
1.1.Dependency Ratio	%	38.2	0.45	46.7	0.51	0	100
1.2.Household head education	%	64.7	0.74	47.2	0.55	0.21	0.71
1.3.% of female household heads	%	13.7	0.27	8.3	0.16	0	100
1.4.% of Household heads with incomplete primary education	%	61.2	0.63	71.0	0.73	0	100
**Average score**			**0.41**		**0.45**		

Source: Fieldwork (2023).

#### 4.2.2. *Livelihood strategies.*

Tables 6 and 7 present aspects of the livelihood strategies of Tay and Thai households, including the level of dependence on agriculture, income diversification, and household members’ participation in non-agricultural activities or working away from home. These are important factors to assess the level of flexibility and adaptability of livelihoods in the context of climate change, especially when agricultural production is increasingly affected by extreme weather, diseases, and market fluctuations. According to Table 6, both ethnic groups are heavily dependent on agriculture. The proportion of households living mainly on crop and livestock farming in the Thai group is 97%, and in the Tay group is 92%. However, the difference lies in the level of livelihood diversification. While 40% of Tay households have at least one member engaged in non-agricultural activities (such as small-scale trading, service work, handicrafts), only 8% of Thai households have this condition. This shows that Tay households are better able to diversify livelihood risks, especially when an industry is in crisis, they still have alternative sources of income. In addition, 25% of Tay households have members working away from home (migrant workers), while in the Thai group this is only 13%. This reflects greater flexibility in mobilizing human resources and expanding income networks in the Tay group. In addition, 33% of Tay households have three or more sources of income, compared to only 9% in the Thai group. In contrast, 68% of Thai households have only one source of income, mostly traditional agriculture, while this rate is 42% in the Tay group.

**Table 6:** Livelihood strategies of Tay and Thai groups.

Livelihood strategies	Percentage	
	Tay group	Thai group
**Main livelihood**		
Agriculture	92	97
Non-agriculture	8	3
**Main agricultural souces**		
Crop cultivation	100	100
Livestock farming	93	86
Forestry	39	71
Forest product harvesting	35	51
Other sources	50	46
Households with primary income source from agriculture	89	97
Households with members working in other places	25	13
Households with members engaged in non-agricultural livelihood	40	8

Source: Fieldwork (2023).

**Table 7:** LVI scores for livelihood strategies.

Livelihood sub-components	Unit	Tay group		Thai group		Min	Max
		Real Value	Standardized Value	Real Value	Standardized Value		
2.1. Percentage of households primarily dependent on crop cultivation/livestock farming/forestry for livelihood	%	94.2	0.89	98.1	0.98	0	100
2.2. Percentage of households with no members working outside the village	%	81.3	0.79	83.2	0.89	0	100
2.3. Percentage of households with no sources of income other than crop cultivation/livestock farming/forestry	%	59.2	0.69	71.2	0.79	0	100
2.4. Average agricultural livelihood diversity index		0.31	0.23	0.41	0.25	0.27	1
**Average score**			**0.76**		**0.85**		

Source: Fieldwork (2023).

The clear difference in livelihood strategies between the two groups is reflected in the scores for this dimension in Table 7. The Thai group has a score of 0.85, significantly higher than the Tay group at 0.76. This is the highest among all LVI components of both groups. This index reflects that the Thai group is facing serious livelihood risks if their main production activities are affected by natural disasters, crop diseases or output crises. Having no alternative livelihoods means that households are vulnerable to income shortages, food insecurity and difficulty in recovering from natural disasters. In general, livelihood diversification not only generates stable income but also serves as a foundation for adaptive capacity. Households with multiple sources of income tend to invest more in education, health and new production techniques, thereby increasing their resilience to risks. On the contrary, the livelihood diversity of the Thai group makes households “tied” to agricultural risks, lacking the ability to compensate when facing adverse fluctuations. Therefore, the results of Tables 6 and 7 not only reflect the current level of vulnerability, but also provide an important basis for proposing policies to support livelihood diversification, especially for ethnic minority communities in mountainous areas with high vulnerability levels such as the Thai group.

#### 4.2.3. *Social and financial networks.*

Tables 8 and 9 present aspects related to access to social and financial networks – factors that play a very important role in determining the resilience of households in the face of environmental shocks and livelihood fluctuations. Social and financial networks include formal and informal relationships such as support from the community, government, social organizations, access to information, as well as access to credit or financial services when needed. According to Table 8, the percentage of households receiving support from the government in the last 3 years in the Thai group is 63.4%, nearly double that of the Tay group (33.1%). Similarly, the percentage of households receiving support from the community in the Thai group is 59.6%, while in the Tay group it is only 12.5%. This shows that Thai households are quite dependent on external support resources, especially in conditions of frequent risks. However, over-reliance on support is also a sign of low self-reliance.

**Table 8:** Social and financial networks.

Social and financial networks	Percentage	
	Tay group	Thai group
Media and comunication		
Television	96.4	51.3
Radio	20.2	5.6
Telephone	89.2	86.1
Internet	11.4	11.1
No means	0	4.8
Received support from the government in the past year	33.1	63.4
Received support from the community in the past month	12.5	59.6
Helped the community in the past month	54.7	59.9
Borrowed money from relatives/friends/neighbors in the past month	25.4	36.6
Lent money to relatives/friends/neighbors in the past month	13.2	3.5
Bank loan	68.0	82.4
Deposit savings at the bank	6.3	1.8

Source: Fieldwork (2023).

**Table 9:** LVI score for social and financial networks.

Social and financial sub-components	Unit	Tay group		Thai group		Min	Max
		Real Value	Standardized Value	Real Value	Standardized Value		
3.1. Percentage of households with no access to information sources	%	0.00	0.00	8.21	0.05	0	100
3.2. Diversity index of information sources		0.43	0.21	0.51	0.41	0.1	1
3.3. Percentage of households receiving assistance from government agencies in the past 12 months	%	36.1	0.38	70.0	0.72	0	100
3.4. Average proportion of receiving: giving (aid)		0.79	0.21	1.21	0.18	0.11	3
3.5. Average proportion of borrowing: lending money within the community		1.31	0.55	1.65	0.49	0.3	3
3.6. Average proportion of borrowing: lending money from/to banks at present		1.75	0.81	1.99	0.78	0.4	3
3.7. Average distance to the town center	km	3.6	0. 4	9.3	0.59	0.9	35
**Average score**		**0.31**		**0.45**			

Source: Fieldwork (2023).

In terms of access to information – a key factor in climate adaptation – only 47.8% of Thai households have a TV or radio that works regularly, compared to 82.3% of Tay households. This significantly affects their ability to obtain weather forecasts, early warnings of natural disasters, or access technical and financial assistance programs. In addition, the average distance from home to the commune or town center for the Thai group is 9.3 km, much higher than the Tay group’s 3.6 km. Longer distances mean higher costs of accessing social services and markets, reducing the ability to network and access livelihood opportunities. In terms of financial access, the Tay group has a higher proportion of households that have borrowed capital from policy banks or people’s credit funds (37.8% compared to 19.2% in the Thai group), indicating a better level of autonomy and proactive access to formal resources. Meanwhile, the Thai group relies more on informal loans or in-kind support from the community, reflecting the lack of stability and sustainability in financial support (Table 8).

Combining the above factors, the LVI index of the Thai group in the social-financial network component reached 0.45, significantly higher than that of the Tay group at 0.31. This difference reflects the higher vulnerability of the Thai group in maintaining social connections and mobilizing resources when natural disasters occur. While the Tay group tends to be more proactive in accessing information and finance, the Thai group is passive, dependent on support and lacking connectivity with social services. Weakness in social and financial connections not only makes households more vulnerable when crises occur, but also reduces the opportunity to recover from risks. This is even more important in the context of climate change, when natural disasters occur more frequently and require communities to be more proactive in accessing information, finance and technology. Therefore, the Thai ethnic group should be prioritized in programs to enhance connectivity, disseminate information, and expand access to formal finance (Table 9).

#### 4.2.4. *Health.*

Table 10 presents the health component indicators in the LVI structure, reflecting the level of health vulnerability and access to health care services of Tay and Thai households. The overall health LVI index of the Thai group is 0.41, significantly higher than that of the Tay group at 0.28. This difference shows that the Thai group has a higher level of health vulnerability, which stems from many factors related to health infrastructure conditions, disease prevalence in the household, and access to health care services. First, the average distance from the household to the nearest health facility is an important factor. In the Thai group, this distance is 18.1 km, while in the Tay group it is only 2.5 km. This is a huge difference, leading to differences in accessibility and response time in emergency situations such as natural disasters, accidents, or epidemics. Difficulty in transportation also increases direct and indirect medical costs, causing many poor households to delay medical care, thereby worsening the health status of household members. Second, the proportion of households with members suffering from chronic diseases or requiring regular care in the Thai group is 48.7%, higher than the Tay group at 33.4%. These households often face high medical costs, long care times, and impacts on the productivity of the remaining members. In particular, when natural disasters occur, interrupted access to medical services will make this group of households vulnerable to livelihood instability. In addition, when asked about access to medical services in the past 12 months, only 41.6% of Thai households said they could go to the doctor when needed, while the rate in the Tay group was 67.3%. Part of the reason is due to geography and cost, but also to language barriers and low levels of education – especially among ethnic minority women. This situation clearly reflects that the Thai group faces not only physical but also social barriers to accessing health care. Finally, the proportion of households with malnourished children in the survey year was also higher among the Thai group (17.5%) than the Tay group (9.3%). This is an important indicator of limitations in access to adequate food and basic health care, which can have long-term impacts on the health and development of future generations.

**Table 10:** LVI score for health.

Health sub-components	Unit	Tay group		Thai group		Min	Max
		Real Value	Standardized Value	Real Value	Standardized Value		
4.1. Percentage of households with family members suffering from chronic illnesses	%	36	0.31	35	0.31	0	100
4.2. Percentage of households with members in need of care	%	41	0.35	42	0.41	0	100
4.3. Percentage of households with family members taking sick leave from work/school in the past 2 weeks due to illness	%	41	0.37	35	0.22	0	100
4.4. Average distance to the nearest healthcare facility	km	2.5	0.05	18	0.70	0.5	38
**Average score**		**0.28**		**0.41**			

Source: Fieldwork (2023).

#### 4.2.5. *Food.*

The Thai group’s LVI for food was 0.47, higher than the Tay group’s 0.43. The Thai group had an average of 5.2 months of food shortage per year, while the Tay group had 3.8 months. In addition, 57.3% of Thai households were partially dependent on forest food sources, compared to only 21.2% of the Tay group. The Thai group also had a higher proportion of households without storage for agricultural products, 63.4%, compared to 38.9% of the Tay group. These factors clearly reflect the precariousness in food security and food storage capacity – especially important in the context of increasingly extreme and unpredictable weather (Table 11).

**Table 11:** LVI score for food.

Food sub-components	Unit	Tay group		Thai group		Min	Max
		Real Value	Standardized Value	Real Value	Standardized Value		
5.1. Percentage of households primarily relying on self-produced food and agricultural products as their main source	%	90.13	0.90	85.62	0.85	0	100
5.2. Average number of months experiencing food shortage.	month	4.01	0.31	5.22	0.35	1	12
5.3. Average diversity index of crops		0.26	0.31	0.29	0.33	0.08	0.5
5.4. Percentage of households mainly dependent on foraging in the forest for their food.	%	8.04	0.08	15.61	0.15	0	100
5.5. Percentage of households without food reserves for the next planting season	%	13.11	0.15	10.11	0.1	0	100
**Average score**			**0.43**		**0.47**		

Source: Fieldwork (2023).

#### 4.5.6. *Water access.*

In both communities, there is relatively favorable access to domestic water sources. Currently, the majority of Thai households use water sourced from watershed forests, which are channeled to the villages. Water is often directed into communal reservoirs for shared usage within the villages. The domestic water sources in Tay villages are more diverse, including natural springs, artesian wells, and drilled wells, but the majority come from natural flowing water sources. Only about 3% of Tay households and 6% of Thai households still use water from natural streams. When using water from communal reservoirs, households tend to transport it back home and store water for gradual consumption, so the distance to the water source is relatively close in both communities. During the dry season, some households mentioned occasional water shortages when the water source in the upper reaches becomes scarce. These households are often located farther from the communal reservoirs of the villages. This issue affects more Thai households (54% of those interviewed) compared to Tay households (32% of those interviewed). The LVI score related to domestic water for Tay people is lower than that of Thai people. Overall, both communities exhibit relatively low vulnerability in this aspect (0.15 for Tay and 0.19 for Thai) (Table 12).

**Table 12:** LVI score for water access.

Water access sub-components	Unit	Tay group		Thai group		Min	Max
		Real Value	Standardized Value	Real Value	Standardized Value		
6.1. Percentage of households primarily using natural sources for domestic water supply	%	2.94	0.03	6.01	0.06	0	100
6.2. Average travel time to the main water source for household use	minutes	9.01	0.45	12.11	0.31	0	60
6.3. Percentage of households with insufficient water supply for year-round use	%	32.22	0.34	54.11	0.52	0	100
**Average score**		**0.15**		**0.19**			

Source: Fieldwork (2023).

#### 4.2.7. *Housing and productive land.*

Table 13 indicates the level of vulnerability in terms of physical conditions – including housing quality and land status – of Tay and Thai households. These are the factors that make up the “sensitivity” component of LVI, which directly affects the ability of households to live and produce agricultural products, especially in the context of climate change increasing the frequency of natural disasters such as flash floods and prolonged heavy rains. The LVI index of housing and land of the Thai ethnic group is 0.11, higher than that of the Tay group at 0.09. Although the difference is not large, it still reflects the disadvantages in physical conditions of Thai households compared to Tay households. Specifically, the proportion of households with non-permanent or degraded houses in the Thai group is 39.2%, significantly higher than 28.9% in the Tay group. These houses are often built with bamboo, rattan, mud walls or old wood, which are easily damaged by heavy rains, strong winds or flash floods. Regarding productive land, an important factor affecting income and food security, the Thai group has a rate of households cultivating on sloping land or land that is easily flooded by heavy rains of 61.4%, compared to 45.3% in the Tay group. Cultivation on vulnerable land not only reduces crop yields but also makes production unstable, easily interrupted by extreme climate events. Moreover, these lands are often difficult to invest in and improve (e.g., terraced fields, drainage systems) due to lack of capital and technical knowledge. Another notable difference is that the average distance from the house to the main productive land of the Thai group is only 1.2 km, while the Tay group is 2.1 km. Although the Thai group has a distance advantage, poor soil conditions and traditional farming practices that are not adapted to climate change make this advantage insufficient to offset the high vulnerability.

**Table 13:** LVI score for housing and productive land.

Housing and productive land sub-components	Unit	Tay People		Thai People		Min	Max
		Real Value	Standardized Value	Real Value	Standardized Value		
7.1. Percentage of households with non-durable housing susceptible to windstorms and hail	%	28.91	0.31	39.23	0.43	0	100
7.2. Percentage of households with low-lying land susceptible to flooding	%	5.61	0.06	9.78	0.09	0	100
7.3. Percentage of households with homes located in areas prone to landslide	%	6.23	0.07	8.43	0.08	0	100
7.4. Average distance to the main agricultural production land	km	6.23	0.69	3.41	0.08	0	25
7.5. Average area of agricultural land susceptible to drought	ha/household	0.35	0.04	0.27	0.04	0	0.5
7.6. Average area of agricultural land susceptible to flooding	ha/household	0.05	0.05	0.09	0.1	0	3
**Average score**		**0.09**		**0.11**			

Source: Fieldwork (2023).

#### 4.2.8. *Disaster and climate change.*

Table 14 reflects the exposure level of Tay and Thai households to natural disasters and the impacts of climate change. This is one of the three main groups of indicators that make up the LVI. This component focuses on measuring the frequency and level of impact of extreme climate events on assets, lives, crops and livelihoods of households in the last 5–7 years. The results show that both groups have very high exposure levels, with the LVI component index being 0.56 for the Tay group and 0.52 for the Thai group. These are the highest values in the entire LVI structure, reflecting the fact that mountainous households are directly, frequently and severely affected by types of natural disasters such as prolonged heavy rains, flash floods, mid-season droughts, landslides and frost. Specifically, 91.2% of Tay households and 89.3% of Thai households said they had suffered property damage due to natural disasters in the past 7 years. Property damage included houses with roofs blown off, walls collapsed, barns damaged, and auxiliary structures such as water tanks and food warehouses being washed away or flooded. Although this rate was similar in the two groups, the level of human loss was significantly different. The rate of households with people injured or killed by natural disasters in the Thai group was 8.9%, nearly double that of the Tay group at 4.1%. The cause may come from the characteristics of the terrain where they live (along streams, steep slopes, high landslide areas), combined with poor housing conditions and weak response capacity. During the investigation, some Thai households said they did not receive early warnings or did not have the conditions to evacuate before the natural disaster occurred. Therefore, priority should be given to establishing an early warning system, training in disaster prevention skills, and planning safe residential areas for ethnic minority communities, especially the Thai ethnic group living in areas at high risk of landslides and flash floods.

**Table 14:** LVI for disaster and CC.

Disaster and CC sub-components	Unit	Tay People		Thai People		Min	Max
		Real Value	Standardized Value	Real Value	Standardized Value		
8.1. Percentage of households experiencing property damage due to natural disasters in the past 7 years	%	95.30	0.85	89.04	0.83	0	100
8.2. Percentage of households with individuals injured or killed due to natural disasters in the past 7 years	%	4.15	0.03	8.94	0.09	0	100
**Average score**		**0.56**		**0.52**			

Source: Fieldwork (2023).

#### 4.2.9. *Comprehensive LVI and LVI- IPCC.*

**Fig 3:**
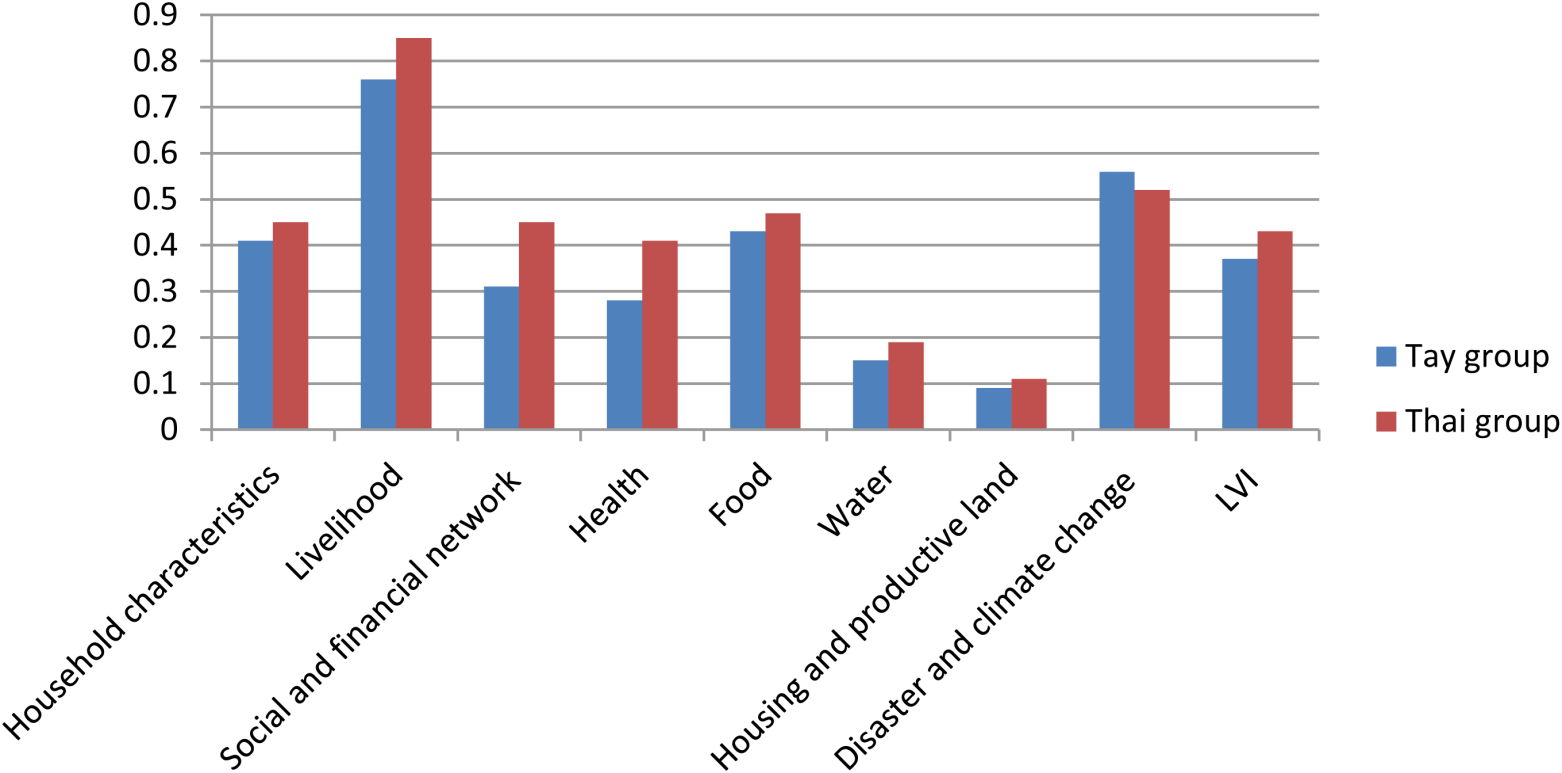
LVI component scores for Tay and Thai groups. Source: Fieldwork (2023).

Table 15 presents the results of LVI calculation using the method of Hahn et al. (2009) for the two ethnic groups Tay and Thai. The results show that the Thai ethnic group has a higher overall LVI index (0.43) than the Tay group (0.37), indicating that the livelihood vulnerability of the Thai people is higher than that of the Tay people in the context of CC. Among the LVI components, the index group with the highest value in both communities is “livelihood strategies”, with 0.76 for the Tay group and 0.85 for the Thai group. This reflects the high dependence of both ethnic minority groups on livelihoods that are vulnerable to CC and the low ability to diversify livelihoods. The Thai group scored higher in most of the components such as social-financial networks (0.45 vs. 0.31), health (0.41 vs. 0.28), food (0.47 vs. 0.43), indicating a more pronounced limitation in adaptive capacity and livelihood conditions. In contrast, the Tay group scored higher in “natural disasters and CC” (0.56 vs. 0.52), indicating that they may be more exposed to natural disasters, but have better coping capacity. It is noteworthy that both groups scored lower in “water” and “housing and productive land”, reflecting a relative stability in basic material conditions. Overall, the results suggest that adaptive capacity – particularly in relation to livelihood diversity, social status, and community support – is a major determinant of differences in vulnerability among ethnic groups. Climate risk mitigation policies should therefore focus on enhancing access to information, vocational training, and social support networks for vulnerable communities such as the Thai ethnic group (Fig 3).

**Table 15:** Comprehensive LVI results.

LVI components	Scores	
	Tay group	Thai group
Household characteristics	0.41	0.45
Livelihood strategies	0.76	0.85
Social and financial network	0.31	0.45
Health	0.28	0.41
Food	0.43	0.47
Water access	0.15	0.19
Housing and productive land	0.09	0.11
Disaster and CC	0.56	0.52
**Comprehensive LVI**	**0.37**	**0.43**

Source: Fieldwork (2023).

Table 16 presents the results of LVI calculations using the IPCC approach for the Tay and Thai groups. Both communities show relatively high levels of vulnerability to CC impacts, with the Thai community being more vulnerable (LVI-IPCC = 0.021) than the Tay community (LVI-IPCC = 0.016). In particular, the level of exposure to/natural disaster/CC impacts does not show significant differences between the two communities. However, the Thai community shows a higher sensitivity to CC impacts (0.295) than the Tay community (0.238). Furthermore, the adaptive capacity of the Thai community in responding to the impacts of CC is also significantly weaker than that of the Tay community (0.513 for the Thai and 0.493 for the Tay). Similar to the comprehensive LVI, the difference in LVI-IPCC index between the two groups reflects the diversity in livelihood vulnerability among ethnic communities, and also shows the important role of social characteristics, livelihood strategies and access to services in shaping the vulnerability to CC of ethnic minority communities.

**Table 16:** LVI-IPCC for Tay and Thai ethnic groups.

Components	Tay group	Thai group
Adaptive capacity	0.493	0.513
Sensitivity	0.238	0.295
Exposure	0.560	0.520
**LVI-IPCC**	**0.016**	**0.021**

Source: Fieldwork (2023).

## 5. Discussions

This paper assesses the livelihood vulnerability to CC among ethnic minority groups in the northern mountainous region of Vietnam. The study uses the Hahn et al. (2009) and IPCC approaches to develop vulnerability indicators. These indicators are also adjusted and aligned to better fit the analytical context in Yen Bai province.

Firstly, the results show that adaptive capacity – particularly in relation to the ability to diversify livelihoods, education, social and financial networks – is a key factor in the differences in livelihood vulnerability among ethnic minority groups in Yen Bai province. Although the exposure to natural hazards is similar between the two groups, the marked differences in adaptive capacity have led to the difference in LVI between the Tay and Thai. This finding is consistent with previous studies on the central role of adaptive capacity in shaping vulnerability to CC [25,22,29,31,33]. According to Hahn et al. (2009), adaptive capacity acts as a balance between exposure and sensitivity, and if enhanced, can significantly reduce livelihood vulnerability. Eriksen et al. (2011) also assert that it is social factors – such as education, access to information, and community support – that determine a community’s adaptive capacity, rather than environmental factors alone. In the Asian context, Gentle and Maraseni (2012) in Nepal and Pandey and Jha (2012) in India both point out that differences in socio-cultural levels among ethnic minority groups are the cause of inequality in the ability to cope with climate risks. Comparing studies in Vietnam, Ha and Nong (2021), and Nguyen et al. (2020) also highlighted that ethnic minority communities with disadvantaged household characteristics, lack of social participation, and limited access to climate information tend to have higher levels of vulnerability, regardless of their actual exposure. This study thus provides further empirical evidence for the view that enhancing adaptive capacity is a key lever to reduce livelihood vulnerability, especially in mountainous ethnic minority areas where natural conditions and climate risks are difficult to control.

Furthermore, all three main aspects of adaptive capacity do not only have a single effect but also have a combined effect, creating a clear difference in the level of livelihood vulnerability among ethnic groups. First, household characteristics reflect background factors such as the education level of the household head, the number of dependents, the gender of the household head, and production experience. The Thai ethnic group has a lower index in this component, indicating that they have a higher proportion of dependents and lower levels of education of household heads, which limits their ability to access information, adapt to new farming techniques and make effective decisions to cope with natural disasters. This finding is consistent with previous studies [25,26,29,35,36], which found that educational capacity and demographics are directly related to the adaptive capacity of farming households. Second, livelihood strategies is the component with the highest value in both survey groups, but significantly higher in the Thai group. This reflects dependence on one main source of income, with less ability to diversify livelihoods. The lack of backup strategies such as off-farm employment, or income from other stable sources, makes these households more vulnerable to crop failures or natural disasters. This is a common weakness among ethnic minority communities, as Gentle and Maraseni (2012) highlighted in their study in Nepal that livelihood diversity is a major cause of reduced resilience in mountain communities. Third, social and financial networks are a proxy for the ability to mobilize external resources, the level of social connections, access to credit, or support from state and non-state organizations. The Thai group has a higher index, which does not mean positivity – but rather reflects a greater dependence on external support in times of difficulty. The Tay group may receive less support, but this may also indicate a higher level of autonomy or stronger internal community connections. Eriksen et al. (2011) and Pandey and Jha (2012) both point out that “self-reliance and endogenous social cohesion” play a significant role in livelihood resilience in the face of climate risks. This suggests that climate adaptation programs need to take an integrated approach, targeting “soft” factors such as skills training, livelihood diversification support, and community connectivity building, rather than focusing solely on physical investments.

Secondly, the study results also show that sensitivity, reflected in factors such as household health, food security, access to water and productive land plays an important role in creating differences in livelihood vulnerability among ethnic minority groups in Yen Bai province. The Thai ethnic group has a higher sensitivity index than the Tay group in most aspects, especially in health and food, indicating a greater dependence on natural conditions and less internal flexibility in the face of climate risks. Specifically, poor quality of health care and health status make Thai people more vulnerable to natural disasters or weather-related epidemics. In addition, lack of food security – reflected in the number of months of hunger in a year and dependence on agricultural production – increases the risk of nutritional and income vulnerability. Limited access to water and productive land, due to topographical conditions or inequitable land tenure, further exposes this group to livelihood insecurity as the environment changes.

This finding is consistent with previous studies on CC vulnerability in developing regions [32,33,37,38]. Hahn et al. (2009) found that sensitivity is a core pillar of the vulnerability construct, where factors such as water, food, and health act as mediators between exposure and actual vulnerability. Alam et al. (2017) found that Bangladeshi coastal communities are highly vulnerable because of their dependence on volatile resources such as freshwater and agricultural land. In their study in the Himalayas, Pandey and Jha (2012) found that households lacking productive land and irrigation systems are more vulnerable to changing weather conditions. In Vietnam, Ha and Nong (2021) asserted that health, food and basic infrastructure factors are the factors that increase the vulnerability of ethnic minority communities in the Northern mountainous region. Therefore, it can be affirmed that the level of sensitivity is not just a secondary factor but a fundamental factor that constitutes the difference in vulnerability between ethnic groups. Groups with limited health conditions, clean water, productive land and food security will always be the most vulnerable group – regardless of the level of exposure to natural hazards. This implies that adaptation strategies and climate risk reduction policies need to be designed not only based on environmental risks, but also to reduce the intrinsic sensitivity of the community.

Thirdly, the findings indicate that both the Tay and Thai ethnic groups have very high levels of exposure to natural disasters and climate change impacts. Although the exposure levels are relatively similar, the Thai group suffered more severe human and crop losses, suggesting that the actual level of vulnerability depends not only on the frequency of risks but also on the ability to respond and prepare. The very high proportion of households that have experienced property damage due to natural disasters in both groups reflects the fact that ethnic minority communities in the highlands are living in high-risk ecological conditions, frequently affected by flash floods, landslides, droughts and erratic weather. This finding is consistent with previous studies on the high exposure levels of mountainous households in Vietnam and the South Asian region [22,25–27,31]. The study by Hahn et al. (2009) in Mozambique using LVI also showed that climate exposure was the most valuable component of overall livelihood vulnerability, especially in riverine, lowland and mountainous communities with difficult terrain. In mountainous Nepal, Pandey and Jha (2012) confirmed that communities living on steep slopes or along streams were at high risk of exposure to landslides, flash floods and extreme rainfall, similar to the situation in Yen Bai province. In Vietnam, Ha and Nong (2021) also recorded high levels of exposure in mountainous districts, in which the majority of surveyed households had suffered damage from floods and droughts in the past 5 years. Studies have shown that when high exposure is combined with low adaptive capacity and high sensitivity, vulnerability will increase significantly. Therefore, although exposure is considered an objective factor and has little direct control, the results of this study suggest that appropriate interventions are needed to minimize the impact of exposure, through measures such as establishing early warning systems, planning residential areas away from dangerous areas, and improving community disaster preparedness skills. The Thai ethnic group, due to their poorly constructed houses, living in steep terrain, and lack of early information, should be prioritized in climate risk reduction intervention programs in mountainous areas.

Overall, the findings of this study reinforce and extend the theoretical utility of the IPCC vulnerability framework (IPCC, 2007), which conceptualizes vulnerability as a function of exposure, sensitivity, and adaptive capacity. While this structure has been widely applied in various settings [25,29,33], our application in the context of upland ethnic minority communities in Vietnam provides important refinements. First, the study empirically demonstrates that adaptive capacity, especially livelihood diversification, community support, and access to information, emerges as a key differentiator of vulnerability even when levels of exposure are similar. This suggests that policy interventions targeting adaptive capacity may yield more immediate impacts on reducing vulnerability in such regions. Second, the results reveal nuanced interactions between sensitivity indicators (e.g., health, access to water, land exposure) and cultural-livelihood practices unique to ethnic groups, an area often overlooked in mainstream applications of the LVI. This supports recent calls for contextualizing vulnerability frameworks to account for sociocultural specificities [25,29,36]. Overall, by linking empirical insights with the conceptual structure introduced earlier, this study not only validates the IPCC-LVI framework in a new geographical and socio-ethnic setting but also contributes to refining the operational understanding of vulnerability in marginalized rural contexts. Future research may build upon this by integrating intersectional and cultural dimensions into vulnerability assessment.

## 6. Conclusions and recommendations

This study has theoretical and practical contributions as well as some specific policy recommendations as follows:

Theoretically, this study contributes to expanding and adjusting the LVI assessment framework of Hahn et al. (2009) and LVI-IPCC in a more suitable direction for the local mountainous context, where livelihoods are closely linked to natural conditions and socio-cultural characteristics. The separation of the demographic profile index into two subgroups: “household characteristics” and “housing and productive land”, and allocating them to two different theoretical components (adaptive capacity and sensitivity) is an academically meaningful adjustment of this study, contributing to better reflecting the multidimensionality and internal differentiation of ethnic minority livelihoods. The study also adds empirical evidence to the IPCC’s theoretical hypothesis that adaptive capacity and sensitivity play an important mediating role in the relationship between exposure and actual vulnerability of the community.

In terms of practice, the study provides reliable quantitative evidence on the differences in livelihood vulnerability among ethnic minority groups in Yen Bai province – a representative area of the northern mountainous region of Vietnam. The analytical results show the need to develop differentiated adaptation policies by population group, prioritizing the enhancement of adaptive capacity through improved education, livelihood diversification and strengthening of social-financial networks. At the same time, the study suggests the possibility of applying LVI or LVI-IPCC as a rapid assessment tool and decision support in climate risk management and socio-economic development planning at the local level. These findings not only serve Yen Bai province but can also be extended to other localities with similar characteristics in terms of terrain, ethnicity and livelihoods.

Based on the analysis results of livelihood vulnerability according to LVI and LVI-IPCC of ethnic minority groups in Yen Bai province, the study gives some specific policy recommendations.

Firstly, it is necessary to strengthen the capacity of ethnic minority households to provide education and skills training. Training programs on climate-resilient agricultural techniques, disaster prevention and household financial management should be organized regularly, in the form of accessible community classes. Integrating knowledge on CC and sustainable livelihoods into agricultural extension or vocational training programs is necessary to raise awareness and decision-making capacity of people. Special attention should be paid to ethnic minority women and youth – groups that play a central role in livelihood development but often do not fully benefit from support policies.

Secondly, it is critical for supporting people to diversify their livelihoods is an important solution to reduce risks and increase resilience to CC. Local authorities need to encourage and support new production models suitable to local conditions such as growing indigenous medicinal plants, adaptive livestock farming, developing community tourism or traditional handicrafts. At the same time, it is necessary to strengthen the connection between people and the market, helping households participate in agricultural value chains with stable output, thereby increasing income and reducing dependence on vulnerable traditional farming.

Thirdly, it is necessary to consolidate and expand community-level social-financial networks such as women’s associations, farmers’ associations, self-managed credit-saving groups, etc. to serve as a foundation to support people in adapting to climate risks. These networks not only act as a place to mobilize financial resources, but also as a channel to share experiences and strengthen community solidarity. At the same time, expanding access to microcredit, agricultural insurance and establishing community-based early warning systems that incorporate indigenous knowledge will help people proactively respond to risks instead of just reacting passively.

Fourthly, focused investment in climate-resilient livelihood infrastructure is an urgent requirement. Projects such as domestic water supply systems, small irrigation systems for production, rural roads to prevent floods or agricultural product storage should be prioritized in vulnerable areas. The implementation of these projects should follow the community-based development method (CBDRM), with direct participation of people in identifying needs, supervising construction and operating after completion, thereby improving the efficiency and sustainability of public investment.

Fifthly, it is necessary to develop and strengthen solutions to reduce the level of sensitivity in livelihood vulnerability, focusing on minimizing weaknesses in the components that make up this factor. First of all, it is necessary to improve the quality and accessibility of health services for people in highland areas, strengthen seasonal disease prevention and regular health care. At the same time, it is necessary to promote local food security through supporting climate-appropriate crop varieties, small-scale garden-pond-barn models and building household-scale agricultural product preservation systems. Small-scale clean water supply systems suitable for mountainous terrain also need to be invested in to ensure that people do not lack water for daily use in the dry season. In addition, there should be policies to support access to and effective use of productive land for ethnic minority households, especially those with small land areas or poor soil fertility. Ensuring stable and equitable land use rights will help people feel secure in investing in long-term livelihoods. Sensitivity indicators should also be integrated into development planning and CC response plans at the commune level, in order to clearly identify the most vulnerable groups and prioritize the allocation of support resources in a more effective, equitable and sustainable manner.

Last but not least, it is important to develop and perfect specific policies for ethnic minority and mountainous areas in the context of CC. The integration of the national target program on sustainable poverty reduction, Program 135 and CC adaptation action plans will help optimize resources and improve implementation efficiency. In addition, using LVI as a decision support tool in the process of socio-economic development planning at the commune level will help orient investment policies according to actual risk levels. Furthermore, establishing a mechanism to monitor and periodically update the level of livelihood vulnerability will help adapt adaptation policies flexibly, effectively and closely follow reality.

## Supporting information

S1 Data (XLSX)

## References

[1] Intergovernmental Panel on Climate Change (IPCC). Climate change 2021: The physical science basis. Contribution of Working Group I to the Sixth Assessment Report of the Intergovernmental Panel on Climate Change. Cambridge: Cambridge University Press; 2021. Available from: https://www.ipcc.ch/report/ar6/wg1/

[2] Djoudi H, Locatelli B, Vaast C, Asher K, Brockhaus M, Basnett Sijapati B. Beyond dichotomies: Gender and intersecting inequalities in climate change studies. Ambio. 2016;45(Suppl 3):248–62. 27878531 10.1007/s13280-016-0825-2 PMC5120018

[3] Hallegatte S, Bangalore M, Bonzanigo L, Fay M, Kane T, Narloch U. Shock waves: Managing the impacts of climate change on poverty. Washington, DC: World Bank; 2016.

[4] Morton JF. The impact of climate change on smallholder and subsistence agriculture. Proc Natl Acad Sci U S A. 2007;104(50):19680–5. doi: 10.1073/pnas.0701855104 18077400 PMC2148357

[5] Revi A, Satterthwaite DE, Aragón-Durand F, Corfee-Morlot J, Kiunsi RBR, Pelling M, et al. Urban areas. In: Field CB, Barros VR, Dokken DJ, et al., editors. Climate change 2014: Impacts, adaptation, and vulnerability. Part A: Global and sectoral aspects. Contribution of Working Group II to the Fifth Assessment Report of the Intergovernmental Panel on Climate Change. Cambridge: Cambridge University Press; 2014. p. 535–612.

[6] Dumenu WK, Obeng EA. Climate change and rural communities in Ghana: Social vulnerability, impacts, adaptations and policy implications. Environ Sci Policy. 2016;55:208–17. doi: 10.1016/j.envsci.2015.10.010

[7] World Bank. Poverty and shared prosperity 2020: Reversals of fortune. Washington, DC: World Bank; 2020.

[8] Oo AT, Huylenbroeck GV, Speelman S. Assessment of climate change vulnerability of farm households in Pyapon District, a delta region in Myanmar. International Journal of Disaster Risk Reduction. 2018;28:10–21. doi: 10.1016/j.ijdrr.2018.02.012

[9] Sarker MNI, Wu M, Alam GMM, Shouse RC. Livelihood Vulnerability of Riverine-Island Dwellers in the Face of Natural Disasters in Bangladesh. Sustainability. 2019;11(6):1623. doi: 10.3390/su11061623

[10] Dumenu WK, Obeng EA. Climate change and rural communities in Ghana: social vulnerability, impacts, adaptations, and policy implications. Environ Sci Policy. 2016;55.

[11] Werners SE, Wise RM, Butler JRA, Totin E, Vincent K. Adaptation pathways: A review of approaches and a learning framework. Environ Sci Policy. 2021;116:266–75.

[12] World Bank. Vietnam: Climate risk country profile. Washington, DC: The World Bank Group; 2022.

[13] Dat TT, Truong DD. Resource management, environment and climate change towards development sustainability in Vietnam from an economic perspective. J Econ Dev. 2020;278(2):2–11.

[14] Ministry of Natural Resources and Environment (MONRE). Climate change and sea level rise scenarios for Vietnam. Hanoi: MONRE; 2020.

[15] Nguyen CT, Ha HN, Tran TTC. Climate change adaptation policies of Vietnam in the Mekong Delta. Russ J Vietn Stud. 2020;4(3):36–45.

[16] Nguyen YTB, Leisz SJ. Determinants of livelihood vulnerability to climate change: Two minority ethnic communities in the northwest mountainous region of Vietnam. Environ Sci Policy. 2021;123:11–20. doi: 10.1016/j.envsci.2021.04.007

[17] Ha VHT, Nong NB. Understanding Livelihood Vulnerability to Climate Change: Evidence from Quang Ninh Province, Vietnam. J Bus Econ Review. 2021;6(2):137–47. doi: 10.35609/jber.2021.6.2(3

[18] Truong DD, Dat TT, Hang ND, Huan LH. Vulnerability Assessment of Climate Change in Vietnam: A Case Study of Binh Chanh District, Ho Chi Minh City. Front Environ Sci. 2022;10. doi: 10.3389/fenvs.2022.880254

[19] Ensor JE, Park SE, Attwood SJ, Kaminski AM, Johnson JE. Can community-based adaptation increase resilience? Clim Dev. 2018;10(2):134–51.

[20] Le TNP. Impact of removing industrial tariffs under the European–Vietnam free trade agreement: A computable general equilibrium approach. J Econ Dev. 2019;21(1):2–17.

[21] Duc Truong D, Tho Dat T, Huy Huan L. Factors Affecting Climate-Smart Agriculture Practice Adaptation of Farming Households in Coastal Central Vietnam: The Case of Ninh Thuan Province. Front Sustain Food Syst. 2022;6. doi: 10.3389/fsufs.2022.790089

[22] Ha BTM, Dung NT. Method to assess the vulnerability of farming households’ livelihoods due to climate change. Vietnam Sci Technol J B. 2018;60(11):20–32.

[23] Que HTH, Thang TN. Diversity of livelihoods and income of farmers in acacia growing areas in the mountainous areas of Thua Thien Hue Province. Hue Univ J Sci Agric Rural Dev. 2020;129(3B):55–68.

[24] Kieu TTH, Nguyen TN, Nguyen THT, Vu THA, Nguyen QT. Indigenous knowledge in climate change adaptation. Econ Dev. 2020;20:45–58.

[25] Pandey R, Jha S. Climate vulnerability index - measure of climate change vulnerability to communities: a case of rural Lower Himalaya, India. Mitig Adapt Strateg Glob Change. 2011;17(5):487–506. doi: 10.1007/s11027-011-9338-2

[26] Gentle P, Maraseni TN. Climate change, poverty and livelihoods: adaptation practices by rural mountain communities in Nepal. Environ Sci Policy. 2012;21:24–34. doi: 10.1016/j.envsci.2012.03.007

[27] Sujakhu NM, Ranjitkar S, He J, Schmidt-Vogt D, Su Y, Xu J. Assessing the Livelihood Vulnerability of Rural Indigenous Households to Climate Changes in Central Nepal, Himalaya. Sustainability. 2019;11(10):2977. doi: 10.3390/su11102977

[28] Tanner T, Lewis D, Wrathall D, Bronen R, Cradock-Henry NA, Huq S. Livelihood resilience in the face of climate change. Nat Clim Change. 2015;14(1):23–6.

[29] Hahn MB, Riederer AM, Foster SO. The Livelihood Vulnerability Index: A pragmatic approach to assessing risks from climate variability and change - A case study in Mozambique. Glob Environ Change. 2009;19(1):74–88.

[30] Nguyen ATT, Nguyen QNT, Au NH. Assessment of livelihood vulnerability to climate change in Phu Hoa district, Phu Yen Province, Vietnam. IOP Conf Ser Earth Environ Sci. 2023;1247(1):012010.

[31] Sallu SM, Twyman C, Stringer LC. Resilient or vulnerable livelihoods? Assessing livelihood dynamics and trajectories in rural Botswana. Ecol Soc. 2010;15(4):3.

[32] Shah KU, Dulal HB, Johnson C, Baptiste A. Understanding livelihood vulnerability to climate change: Applying the livelihood vulnerability index in Trinidad and Tobago. Geoforum. 2013;47:125–37. doi: 10.1016/j.geoforum.2013.04.004

[33] Hedlund J, Fick S, Carlsen H, Benzie M. Quantifying transnational climate impact exposure: New perspectives on the global distribution of climate risk. Global Environ Change. 2018;52:75–85. doi: 10.1016/j.gloenvcha.2018.04.006

[34] Etwire PM, Al-Hassan RM, Kuwornu JKM, Osei-Owusu Y. Application of Livelihood Vulnerability Index in Assessing Vulnerability to Climate Change and Variability in Northern Ghana. J Environ Earth Sci. 2013;3(2):157–70.

[35] Ahsan MdN, Warner J. The socioeconomic vulnerability index: A pragmatic approach for assessing climate change led risks–A case study in the south-western coastal Bangladesh. Int J Disaster Risk Reduct. 2014;8:32–49. doi: 10.1016/j.ijdrr.2013.12.009

[36] IPCC. Climate Change 2001: Impacts, Adaptation, and Vulnerability. Cambridge: Cambridge University Press; 2001.

[37] Shen J, Duan W, Wang Y, Zhang Y. Household Livelihood Vulnerability to Climate Change in West China. Int J Environ Res Public Health. 2022;19(1):551. doi: 10.3390/ijerph19010551 35010816 PMC8744803

[38] Alam GM, Alam K, Mushtaq S. Climate change perceptions and local adaptation strategies of hazard-prone rural households in Bangladesh. Clim Risk Manag. 2017;17:52–63.

[39] Shah KU, Dulal HB, Johnson C, Baptiste A. Understanding livelihood vulnerability to climate change: Applying the livelihood vulnerability index in Trinidad and Tobago. Geoforum. 2013;47:125–37. doi: 10.1016/j.geoforum.2013.04.004

[40] Hair JF, Hult GTM, Ringle CM, Sarstedt M. A primer on partial least squares structural equation modeling (PLS-SEM). 2nd edition. Thousand Oaks, CA: Sage; 2019.

[41] General Statistics Office. Provincial data annual report of Vietnam. Hanoi: GSO; 2022.

[42] Yen Bai People Committee. Overview about Yen Bai province 2022 [Internet]. 2022 Available from: https://yenbai.gov.vn/Pages/Vi-Tri-dia-Ly.aspx?ItemID=7&l=vitridialy

[43] Nguyen TB. Livelihood vulnerability and choice of climate change adaptation solutions of ethnic minority communities in Mo Vang commune, Van Yen, Yen Bai [MSc thesis]. Hanoi: Earth Science; 2019.

[44] Thanh CP. Impact of price policy on production choices of farmers in Van Chan district, Yen Bai province [doctoral thesis]. Hanoi: Univ Econ Bus Admin; 2021.

